# The Predictive Value of Systemic Inflammatory Markers, the Prognostic Nutritional Index, and Measured Vessels’ Diameters in Arteriovenous Fistula Maturation Failure

**DOI:** 10.3390/life12091447

**Published:** 2022-09-18

**Authors:** Réka Kaller, Emil Marian Arbănași, Adrian Vasile Mureșan, Septimiu Voidăzan, Eliza Mihaela Arbănași, Emőke Horváth, Bogdan Andrei Suciu, Ioan Hosu, Ioana Halmaciu, Klara Brinzaniuc, Eliza Russu

**Affiliations:** 1 Clinic of Vascular Surgery, Mureș County Emergency Hospital, 540136 Târgu-Mureș, Romania; 2 Doctoral School of Medicine and Pharmacy, University of Medicine, Pharmacy, Science and Technology “George Emil Palade” of Târgu-Mureș, 540139 Târgu-Mureș, Romania; 3 Department of Surgery, University of Medicine, Pharmacy, Science and Technology “George Emil Palade” of Târgu-Mureș, 540139 Târgu-Mureș, Romania; 4 Department of Epidemiology, University of Medicine, Pharmacy, Science and Technology “George Emil Palade” of Târgu-Mureș, 540139 Târgu-Mureș, Romania; 5 Faculty of Pharmacy, University of Medicine, Pharmacy, Science and Technology “George Emil Palade” of Târgu-Mureș, 540139 Târgu-Mureș, Romania; 6 Department of Pathology, University of Medicine, Pharmacy, Science and Technology “George Emil Palade” of Târgu-Mureș, 540142 Târgu-Mureș, Romania; 7 Department of Anatomy, University of Medicine, Pharmacy, Science and Technology “George Emil Palade” of Târgu-Mureș, 540142 Târgu-Mureș, Romania; 8 Department of Nephrology, Mures County Emergency Hospital, 540136 Târgu-Mureș, Romania

**Keywords:** arteriovenous fistula, brachio-cephalic AVF, radio-cephalic AVF, end-stage renal disease dialysis, maturation, NLR, PLR, SII

## Abstract

Background: An arteriovenous fistula (AVF) is the first-line vascular access pathway for patients diagnosed with end-stage renal disease (ESRD). In planning vascular access, it is necessary to check the diameters of the venous and arterial components for satisfactory long-term results. Furthermore, the mechanism underlying the maturation failure and short-term patency in cases of AVFs is not fully known. This study aims to verify the predictive role of inflammatory biomarkers (the neutrophil to lymphocyte ratio (NLR), platelet to lymphocyte ratio (PLR), systemic inflammatory index (SII), and C-reactive protein (CRP)), Ca-P product, the prognostic nutritional index (PNI), and the diameters of the venous and arterial components in the failure of AVF maturation. Methods: The present study was designed as an observational, analytical, and retrospective cohort study with a longitudinal follow-up, and included all patients with a diagnosis of ESRD that were admitted to the Vascular Surgery Clinic of the Targu Mures Emergency County Hospital, Romania, between January 2019 and December 2021. Results: The maturation of AVF at 6 weeks was clearly lower in cases of patients in the high-NLR (31.88% vs. 91.36%; *p* < 0.0001), high-PLR (46.94% vs. 85.55%; *p* < 0.0001), high-SII (44.28% vs. 88.89%; *p* < 0.0001), high-CRP (46.30% vs. 88.73%; *p* < 0.0001), high-Ca-P product (40.43% vs. 88.46%; *p* < 0.0001), and low-PNI (34.78% vs. 91.14%; *p* < 0.0001) groups, as well as in patients with a lower radial artery (RA) diameter (40% vs. 94.87%; *p* = 0.0009), cephalic vein (CV) diameter (44.82% vs. 97.14%; *p* = 0.0001) for a radio-cephalic AVF (RC-AVF), and brachial artery (BA) diameter (30.43% vs. 89.47%; *p* < 0.0001) in addition to CV diameter (40% vs. 94.59%; *p* < 0.0001) for a brachio-cephalic AVF (BC-AVF), respectively. There was also a significant increase in early thrombosis and short-time mortality in the same patients. A multivariate analysis showed that a baseline value for the NLR, PLR, SII, CRP, Ca-P product, and PNI was an independent predictor of adverse outcomes for all of the recruited patients. Furthermore, for all patients, a high baseline value for vessel diameter was a protective factor against any negative events during the study period, except for RA diameter in mortality (*p* = 0.16). Conclusion: Our findings concluded that higher NLR, PLR, SII, CRP, Ca-P product, and PNI values determined preoperatively were strongly predictive of AVF maturation failure, early thrombosis, and short-time mortality. Moreover, a lower baseline value for vessel diameter was strongly predictive of AVF maturation failure and early thrombosis.

## 1. Introduction

An arteriovenous fistula (AVF) is the first-line vascular access pathway for patients diagnosed with end-stage renal disease (ESRD), with a lower rate of complications and superior patency compared to an arteriovenous graft (AVG) and a central venous dialysis catheter (CVC) [1,2,3,4,5]. For efficient hemodialysis, the vascular access path must be optimal, ensuring a minimum flow of 300 mL/min, being cannulated with two needles, and presenting prolonged patency [6,7].

Although an AVF is the vascular access pathway recommended by the European Society of Vascular Surgery (ESVS) guide [6] to be used, an AVF must be matured. Regarding maturation, an AVF must ensure a sufficient lumen and flow at the level of the venous component to be located superficially for easy and efficient cannulation [8,9,10]. Another important factor in the long-term quality of vascular access is the time of performing an AVF; patients who are prepared for vascular access in terms of time report a higher rate of maturation with better long-term results [11] compared to those who occur late and require the initiation of hemodialysis at the level of a CVC until the maturation of an AVF [12,13,14].

In planning vascular access, it is necessary to check the diameters of the venous and arterial components for satisfactory long-term results. Thus, the ESVS guidelines recommend a minimum diameter of 2 mm for both components for a radio-cephalic AVF (RC-AVF) and a minimum diameter of 3 mm for both components to create a brachio-cephalic AVF (BC-AVF) [6].

The mechanism underlying the maturation failure and short-term patency in cases of AVFs is not fully known. The link between systemic inflammation and short-term AVF failure has been recently studied [15,16,17,18,19]. Among the recently most studied inflammatory markers in the literature, we mention the neutrophil to lymphocyte ratio (NLR) and the platelet to lymphocyte ratio (PLR) as having predictive roles in the negative evolution of patients with a cardiovascular pathology [20,21,22,23,24,25,26,27,28] and patients with chronic kidney disease (CKD), respectively [17,18,29,30,31,32,33]. Another typical inflammatory marker is the systemic inflammatory index (SII), which predicts mortality and poor oncological pathology outcomes [34,35,36].

Nutritional evaluations, in conjunction with systemic inflammatory biomarkers, provide valuable information on the status of ESKD patients. The prognostic nutritional index (PNI) is a simple instrument derived from serum albumin levels and the total lymphocyte count, which represents the condition of systemic inflammation and protein synthesis deficiency in the status of ESKD [37]. Recent studies have shown that this marker can predict the unfavorable progression of individuals with renal disease [38,39,40] as well as the risk of early postoperative renal failure in oncological patients [41,42].

This study aims to verify the predictive role of inflammatory biomarkers (the NLR, PLR, SII, and CRP), Ca-P product, the PNI, and the diameters of venous and arterial components in the failure of AVF maturation.

## 2. Materials and Methods

### 2.1. Study Design

The present study was designed as an observational, analytical, and retrospective cohort study with a longitudinal follow-up. It included all patients with a diagnosis of ESRD that were admitted to the Vascular Surgery Clinic of the Târgu-Mureș Emergency County Hospital, Romania, between January 2019 and December 2021. The exclusion criteria were as follows: ESRD patients who had already had an AVF, an active tumoral status, sepsis, hematological diseases, a personal history of a major surgery in the previous six months, and autoimmune diseases.

Patients included in the study were initially divided into groups depending on their poor AVF maturation status at 6 weeks: “Maturation” and “Non-Maturation”. An ideal cut-off value for the NLR, PLR, SII, CRP, Ca-P product, PNI, and vessel diameters versus maturation was used to calculate each patient’s six-week early thrombosis rate and mortality rate.

### 2.2. Data Collection

The patients’ demographic data were extracted from the hospital’s electronic database. We searched for the following comorbidities in the medical history: arterial hypertension (AH), atrial fibrillation (AF), chronic heart failure (CHF), ischemic heart disease (IHD), myocardial infarction (MI), type 2 diabetes (T2D), cerebrovascular accident (CVA), peripheral arterial disease (PAD), tobacco use, and obesity.

### 2.3. Preoperative Workup and AVF Technique

Physical and Doppler ultrasound exams as well as blood tests (hemoglobin, hematocrit, neutrophil count, lymphocyte count, monocyte count, platelet count, glucose level, cholesterol, and triglyceride level) were conducted before surgery. The NLR, PLR, SII, Ca-P product, and PNI were calculated using the equations below:

$$\mathrm{NLR}=\frac{\mathrm{total}\;\mathrm{number}\;\mathrm{of}\;\mathrm{neutrophils}}{\mathrm{total}\;\mathrm{number}\;\mathrm{of}\;\mathrm{lymphocytes}}$$

$$\mathrm{PLR}=\frac{\mathrm{total}\;\mathrm{number}\;\mathrm{of}\;\mathrm{platelets}}{\mathrm{total}\;\mathrm{number}\;\mathrm{of}\;\mathrm{lymphocytes}}$$

$$\mathrm{SII}=\frac{\mathrm{total}\;\mathrm{number}\;\mathrm{of}\;\mathrm{neutrophils}\times \mathrm{total}\;\mathrm{number}\;\mathrm{of}\;\mathrm{platelets}}{\mathrm{total}\;\mathrm{number}\;\mathrm{of}\;\mathrm{lymphocytes}}$$

Ca-P Product = calcium level (mg/dL) × phosphorous level (md/dL)

PNI = [10 × serum albumin (g/dL)] + [0.005 × total number of lymphocytes]

RC-AVFs and BC-AVFs were created. First, clinically palpable pulses were checked, followed by an ultrasonography examination. The first option was always an RC-AVF. If any of the active component’s diameter was lower than 1.7 mm, a vein had thrombosis stigmata, or an artery appeared heavily calcified, a decision was made to choose the cubital fossa site as the recipient for a BC-AVF.

### 2.4. AVF Maturation

A clinical examination was undertaken for the initial AVF, and the presence of a palpable thrill at the level of the anastomosis was examined for the proper length along the path of the vein, which must be located rather superficially and can be punctured with two needles. An auscultatory continuous audible bruit was registered. Subsequently, the “rule of 6” was verified by ultrasonography, meaning a vein with a minimum diameter of 6 mm, at a maximum depth of 6 mm, and with a minimum flow of 600 mL/min [6].

### 2.5. Study Outcomes

The primary endpoints were the six-week maturation rate, early thrombosis, and mortality. The secondary endpoint was the overall maturation rate after a single assisted maturation intervention. The primary outcomes were stratified for the optimal NLR, PLR, SII, CRP, Ca-P product, PNI, and vessel diameter cut-off value at baseline, and overall outcomes were stratified by AVF type.

### 2.6. Statistical Analysis

SPSS for Mac OS version 28.0.1.0 was used for the statistical analysis (SPSS, Inc., Chicago, IL, USA). Chi-square tests were used to assess the associations of the NLR, PLR, SII, CRP, Ca-P product, PNI, and vessel diameters with category factors, while Student’s *t*-tests or Mann–Whitney U tests were used to assess differences in the continuous variables. To assess the predictive power and establish cut-off NLR, PLR, SII, CRP, Ca-P product, PNI, and vessel diameter values, a receiver operating characteristic (ROC) curve analysis was utilized. The receiver operating characteristic (ROC) curve analysis was utilized to determine the appropriate NLR, PLR, SII, CRP, Ca-P product, PNI, and vessel diameter cut-off values based on Youden’s index (Youden’s index = Sensitivity + Specificity1, ranging from 0 to 1). To identify independent predictors of maturation, early thrombosis, and mortality, a multivariate logistic regression analysis using variables with *p* < 0.1 was undertaken.

## 3. Results

During the studied period, 125 patients with predialysis ESRD were admitted for an AVF procedure. Of the patients, 76 were male (60.80%) and the mean age was 61.64 ± 13.81 (21–84). As for the performed surgical procedures, an RC-AVF was chosen in 64 cases (51.2%) and a BC-AVF was chosen in 61 cases (48.8%). In the first 6 weeks, 22 AVFs suffered early thrombosis and 10 patients died. The 22 thrombosed AVFs were surgically revised as follows: a successful thrombectomy was performed on 16, while the other 6 patients required an additional enlargement angioplasty using bovine pericardium at the anastomosis level to achieve a palpable thrill. Of these patients, 13 reached maturation in the end, while 9 required the performance of a novel AVF. The rest of the comorbidities and laboratory data are presented in Table 1.

Patients whose AVFs failed to mature during the first 6 weeks were older patients (*p* = 0.03). Additionally, in terms of comorbidities, patients in the Non-Maturation group had higher incidences of both CHF (*p* = 0.0004) and T2D (*p* = 0.0001). Regarding the laboratory findings, patients in the Non-Maturation group had higher neutrophil (*p* < 0.0001), serum phosphorous (*p* < 0.0001), Ca-P product (*p* < 0.0001), CRP (*p* < 0.0001), NLR (*p* < 0.0001), PLR (*p* < 0.0001), and SII (*p* < 0.0001) values as well as lower lymphocyte (*p* < 0.0001), serum albumin (*p* < 0.0001), serum calcium (*p* < 0.0001), and PNI (*p* < 0.0001) values. Regarding vessel diameter, in the Non-Maturation group lower vessel diameters were found for both for RC-AVFs (radial artery (*p* < 0.0001), cephalic vein (*p* < 0.0001)) and BC-AVFs (brachial artery (*p* < 0.0001), cephalic vein (*p* < 0.0001)). Moreover, there were higher incidences of early thrombosis (*p* = 0.0001) and mortality (*p* = 0.008) (Table 1).

The statistics show no significant differences in terms of six-week maturation, early thrombosis, and mortality in the two types of AVF, as seen in Table 2. However, the overall maturation rate was higher in the BC-AVF group (95.08% vs. 79.68%; *p* = 0.01).

**Figure 1:**
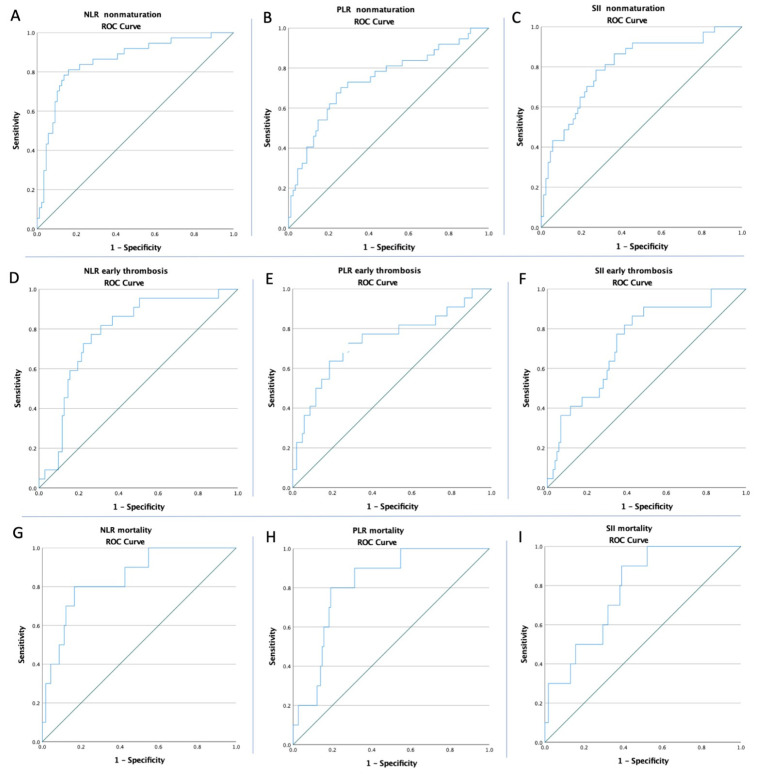
ROC curve analysis (**A**) for the NLR concerning non-maturation, (**B**) for the PLR concerning non-maturation, and (**C**) for the SII concerning non-maturation; (**D**) for the NLR concerning early thrombosis, (**E**) for the PLR concerning early thrombosis, and (**F**) for the SII concerning early thrombosis; and (**G**) for the NLR concerning mortality, (**H**) for the PLR concerning mortality, and (**I**) for the SII concerning mortality.

**Figure 2:**
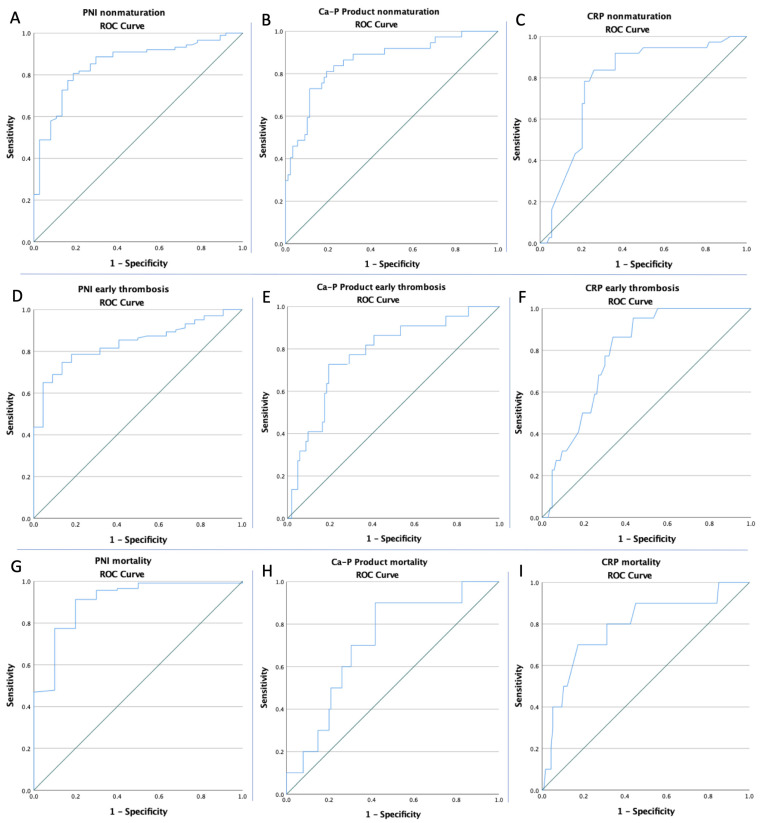
ROC curve analysis (**A**) for the PNI concerning non-maturation, (**B**) for Ca-P product concerning non-maturation, and (**C**) for CRP concerning non-maturation; (**D**) for the PNI concerning early thrombosis, (**E**) for Ca-P product concerning early thrombosis, and (**F**) for CRP concerning early thrombosis; and (**G**) for the PNI concerning mortality, (**H**), for Ca-P product concerning mortality, and (**I**) for CRP concerning mortality.

**Figure 3:**
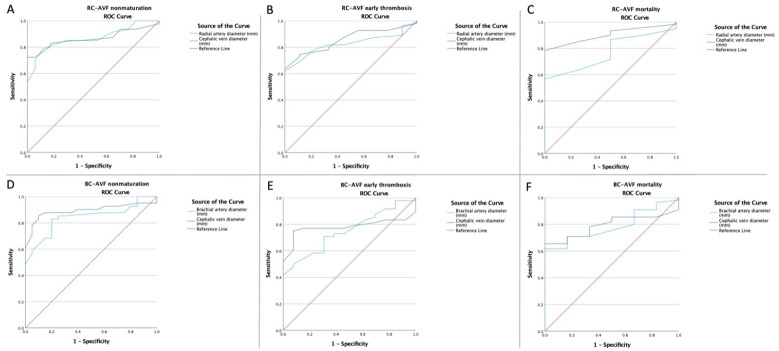
ROC curve analysis (**A**) for the radial artery and cephalic vein diameters concerning non-maturation in RC-AVF patients, (**B**) for the radial artery and cephalic vein diameters concerning early thrombosis in RC-AVF patients, and (**C**) for the radial artery and cephalic vein diameters concerning mortality in RC-AVF patients; (**D**) for the brachial artery and cephalic vein diameters concerning non-maturation in BC-AVF patients, (**E**) for the brachial artery and cephalic vein diameters concerning early thrombosis in BC-AVF patients, and (**F**) for the brachial artery and cephalic vein diameters concerning mortality in BC-AVF patients.

ROC curves for the NLR, PLR, SII, CRP, Ca-P product, PNI, and vessel diameters were created to determine whether the baselines of these biomarkers were predictive of non-maturation, early thrombosis, and mortality in all of the patients (Figure 1, Figure 2 and Figure 3). The optimal cut-offs, obtained from Youden’s index, the areas under the curve (AUCs), and the predictive accuracies of the ratios and vessel diameters are listed in Table 3.

Depending on the optimal cut-off value according to the ROC, the outcomes were further analyzed after dividing the patients into paired groups, as seen in Table 4.

There was a higher incidence in all of the outcomes studied in the high-ratio inflammatory markers and Ca-P product groups, and a lower incidence for all of the outcomes evaluated in the high-PNI and high-vessel-diameter group, except for the RA diameter in regard to mortality in RC-AVFs (*p* = 0.10).

The multivariate analysis showed that a baseline value of NLR > 4.90 predicts AVF maturation failure (OR: 22.65; 95% CI: 8.32–61.67; *p* < 0.001) and early thrombosis (OR: 9.57; 95% CI: 3.21–28.45; *p* < 0.001), whereas an NLR > 5.83 predicts short-term mortality (OR: 19.0; 95% CI: 3.75–96.27; *p* < 0.001). Furthermore, a PLR > 172.29 value is a predictor of maturation failure (OR: 6.68; 95% CI: 2.85–15.63; *p* < 0.001), a PLR > 181.72 is a predictor of early thrombosis (OR: 6.80; 95% CI: 2.42–19.09; *p* < 0.001), and a PLR > 212.89 is an independent predictor of short-term mortality (OR: 16.9; 95% CI: 3.35–85.24; *p* < 0.001). A preoperative value of SII > 954.54 is also a predictor of maturation failure (OR: 9.66; 95% CI: 3.88–24.07; *p* < 0.001), an SII > 859.22 is a predictor of early thrombosis (OR: 7.08; 95% CI: 2.23–22.46; *p* < 0.001), and an SII > 949.71 is an independent predictor of short-term mortality (OR: 14.0; 95% CI: 1.71–114.28; *p* = 0.01). Additionally, high values of CRP and Ca-P product are negative prognostic factors for all of the recorded outcomes (*p* < 0.001, *p* < 0.001, and *p* = 0.003/*p* = 0.01). High PNI levels, on the other hand, are protective factors against adverse events (*p* < 0.0001). Moreover, the presence of CHF and T2D was an independent predictor for non-maturation and early thrombosis. Furthermore, for all patients, a high baseline value for vessel diameter was a protective factor against any negative event during the studied period, except for the RA diameter in mortality (*p* = 0.16) (Table 5).

## 4. Discussion

This research included 125 patients with predialysis ESRD. These patients had 64 RC-AVF and 61 BC-AVF procedures performed. The predictive role of systemic inflammatory markers such as the NLR, PLR, and SII, as well as the diameter of the venous and arterial components regarding the six-week maturation of AVFs, were studied. The study’s most important findings emphasize the predictive role of inflammatory indicators and the importance of vascular diameter for AVF maturation failure.

Numerous studies have examined the relationship between systemic inflammation and AVF failure [43,44,45]. Among the biomarkers studied with a role in predicting AVF thrombosis and maturation failure, we list interleukin-6 (IL-6), tumor necrosis factor-α (TNF-α), and C-reactive protein (CRP) [46,47,48,49].

Similar to this study, Yaprak et al. found that high NLR (HR: 2.72; 95% CI: 1.05–7.02; *p* = 0.03) and PLR (HR: 2.86; 95% CI: 1.11–7.38; *p* = 0.03) values are associated with all causes of mortality, but only the PLR (HR: 4.41; 95% CI: 1.37–14.17; *p* = 0.01) is an independent prognostic factor in multivariate analysis [49]. Moreover, Wongmahisorn demonstrated that high values, both preoperative (OR: 5.46; 95% CI: 3.15–9.48) and postoperative (OR: 7.19; 95% CI: 4.12–12.5), of the NLR are an associated factor for early AVF failure [17].

In a paper published by Zhu et al., which analyzed the association of high NLR and PLR values with balloon post-angioplasty restenosis in AVF stenosis in a group of 114 patients, a PLR > 187.86 before intervention has been associated with post-angioplasty restenosis [50]. The prognostic relevance of NLR and PLR in chronic renal disease has been described in various articles in the literature [51,52,53,54,55,56,57,58,59,60,61].

In terms of vessel diameter, there are mixed results in the literature. Numerous pieces of research have established and affirmed the predictive function of arterial and venous components’ diameters in long-term fistula maturation and survival [62,63,64]; however, some investigations have not found a well-defined connection between arterial diameter and the maturation as well as patency of AVFs [65,66,67].

Therefore, in a comprehensive study, Kordzaev et al. revealed that a minimum diameter of 2 mm for the radial artery and the cephalic vein in conducting an RC-AVF is ideal for long-term development and usefulness [62]. Furthermore, Mendez et al. observed that with a venous diameter of 2 mm they had 16% successful maturation of AVFs, compared to 76% effective maturation in patients with a venous component diameter > 2 mm [63].

In their brief research, Parmar et al. reported that in a group of 21 patients a radial artery diameter greater than 1.5 mm was related to 100% patency at 12 weeks postoperatively (*p* < 0.01) [64]. Wong et al. discovered no difference in the diameter of the venous component between groups with matured AVFs and those with non-matured AVFs [65]. In a paper published by Wlimink et al., which included 803 patients with AVFs, the authors reported that vessel diameter is a weak predictor of AVF functionality [66]. In another 96-patient prospective piece of research, Zadeh et al. discovered no statistical relevance between vessel diameter and AVF maturation [67].

According to the findings of Barutcu Atas et al., a baseline value of PNI < 39 was correlated with mortality in a retrospective study on 359 patients over the age of 80 with CKD stage 3–4 [68]. Furthermore, in a group of 1988 patients with stable coronary arteries, Wada et al. established the involvement of the PNI in the development of significant adverse cardiac events [69].

In terms of inflammatory markers, NLR, PLR, SII, CRP, and Ca-P product values over the baseline are independent predictors of maturation failure, early thrombosis, and short-term mortality, as seen in Table 5, according to the multivariate analysis. Additionally, a high baseline value of the PNI was a protective factor for any negative events during the studied period.

Regarding RC-AVFs, a diameter of RA > 2.25 mm is a protection factor against maturation failure (*p* < 0.001), and an RA > 2.35 mm is a protection factor against early thrombosis (*p* = 0.009) but not against short-term mortality (*p* = 0.16). Additionally, a CV diameter > 2.55 mm is a protection factor against maturation failure (*p* < 0.001), a CV > 2.35 mm is a protection factor against early thrombosis (*p* = 0.004), and a CV > 2.15 mm is a protection factor against short-term mortality (*p* = 0.04).

Regarding BC-AVFs, a BA diameter > 2.95 mm is a protection factor against maturation failure (*p* < 0.001) as well as early thrombosis (*p* = 0.01), and a BA > 2.70 mm is a protection factor against short-term mortality (*p* = 0.02). Additionally, a CV diameter > 2.70 mm is a protection factor against maturation failure (*p* < 0.001) and early thrombosis (*p* = 0.001), and a CV > 2.45 mm is a protection factor against short-term mortality (*p* = 0.02).

Despite these results, this study had some limitations. First, it was a retrospective study with a small number of patients from a single center, in which short-term outcomes were monitored. Secondly, the abundance of exclusion criteria additionally reduced the batch of patients. In the future, we recommend conducting a prospective, multicenter study with long-term outcome monitorization and the recording of the causes of primary patency failure. Another limitation was the non-recorded or -assessed impacts of chronic medications used before admission (such as corticosteroids and anti-inflammatory drugs) on inflammatory biomarkers. Furthermore, additional research is necessary to support our findings.

## 5. Conclusions

Our findings concluded that higher preoperative NLR, PLR, SII, CRP, and Ca-P product values determined before operations strongly predict AVF maturation failure, early thrombosis, and short-time mortality. Secondly, the small preoperative diameters of RA, BA, and CV, as partners in the RC-AVF and BC-AVF anastomoses, strongly predicted AVF maturation failure, early thrombosis, and short-time mortality. Moreover, a higher PNI value was a protective factor for any negative event during the studied period. Given the accessibility and low cost of the ratios and of determining vessel diameters, they can be considered for preoperative risk group stratification, better patient management, and developing predictive patterns.

## Figures and Tables

**Table 1:** Demographic, clinical, and laboratory data, type of AVF, and outcomes of all patients included in the analysis and of the two sub-groups evaluated according to Maturation and Non-Maturation.

Variables	All Patients *n* = 125	Maturation *n* = 88	Non-Maturation *n* = 37	*p*-Value (OR; CI 95%)
**Mean age ± SD (min–max)**	61.64 ± 13.81 (21–84)	60.32 ± 14.82 (21–84)	64.75 ± 10.58 (37–84)	0.03
**Male sex no. (%)**	76 (60.80%)	55 (62.5%)	21 (56.76%)	0.54 (0.78; 0.36–1.71)
**Comorbidities and Risk Factors**				
**AH, no. (%)**	102 (81.6%)	71 (80.68%)	31 (83.78%)	0.68 (1.23; 0.44–3.43)
**AF, no. (%)**	34 (27.2%)	22 (25%)	12 (32.43%)	0.39 (1.44; 0.62–3.33)
**CHF, no. (%)**	47 (37.6%)	24 (27.27%)	23 (62.16%)	0.0004 (4.38; 1.94–9.88)
**IHD, no. (%)**	83 (66.4%)	55 (62.5%)	28 (75.68%)	0.15 (1.86; 0.78–4.43)
**MI, no. (%)**	55 (44%)	36 (40.91%)	19 (51.35%)	0.28 (1.52; 0.70–3.30)
**T2D, no. (%)**	52 (41.6%)	26 (29.55%)	26 (70.27%)	0.0001 (5.63; 2.43–13.06)
**CVA, no. (%)**	40 (32%)	25 (28.41%)	15 (40.54%)	0.18 (1.78; 0.76–3.83)
**PAD, no. (%)**	32 (25.6%)	20 (22.73%)	12 (32.43%)	0.25 (1.63; 0.69–3.81)
**Tobacco, no. (%)**	43 (34.4%)	27 (30.68%)	16 (43.24%)	0.11 (1.90; 0.85–4.25)
**Obesity, no. (%)**	27 (21.6%)	21 (23.86%)	6 (16.22%)	0.34 (0.61; 0.22–1.68)
**Laboratory Data**				
**Hemoglobin g/dL, median [Q1–Q3]**	13.79 [12.89–14.97]	13.88 [12.89–14.97]	13.67 [12.5–14.6]	0.23
**Hematocrit %, median [Q1–Q3]**	42.11 [39.1–45]	42.45 [39.11–45.21]	41.43 [37–44.5]	0.13
**Neutrophils × 10^3^/µL, median [Q1–Q3]**	5.43 [3.92–7.04]	4.9 [3.74–6.5]	6.56 [5.43–8.66]	<0.0001
**Lymphocytes × 10^3^/µL, median [Q1–Q3]**	1.38 [1.05–1.89]	1.56 [1.12–2.07]	1.07 [0.88–1.3]	<0.0001
**Monocyte × 10^3^/µL, median [Q1–Q3]**	0.66 [0.51–0.95]	0.66 [0.55–0.92]	0.69 [0.45–0.97]	0.44
**PLT × 10^3^/µL, median [Q1–Q3]**	219 [170–270]	212.5 [166.5–272.5]	227 [173–265]	0.21
**Glucose mg/dL, median [Q1–Q3]**	107 [91.9–143.5]	102.85 [91.57–144.95]	110 [92.9–134]	0.32
**Cholesterol mg/dL, median [Q1–Q3]**	171.8 [145.4–214.9]	170.8 [143.9–219.45]	187.2 [154–208.4]	0.32
**Triglyceride mg/dL, median [Q1–Q3]**	117.6 [87.3–159.6]	121.1 [88.87–165]	107 [84.1–137.1]	0.21
**GFR (** **mL/min/1.73 m^2^), median [Q1–Q3]**	10.19 [5.88–21.59]	11.16 [5.94–20.03]	9.25 [5.26–21.81]	0.29
**Serum albumin mg/dL, median [Q1–Q3]**	3.57 [3.13–3.96]	3.78 [3.45–4.1]	2.93 [2.63–3.21]	<0.0001
**Serum calcium mg/dL, median [Q1–Q3]**	8.62 [7.89–9.26]	8.86 [8.22–9.50]	7.90 [6.77–8.82]	<0.0001
**Serum phosphorous mg/dL, median [Q1–Q3]**	4.76 [3.32–5.74]	3.80 [3.18–5.06]	6.74 [5.77–7.83]	<0.0001
**PNI, median [Q1–Q3]**	43.10 [37–46.85]	46.25 [41.78–49.55]	34.55 [32.3–37.2]	<0.0001
**Ca-P product, median [Q1–Q3]**	39.34 [29.32–50.66]	32.51 [27.30–42.93]	51.48 [48.16–59.55]	<0.0001
**CRP mg/dL, median [Q1–Q3]**	2.02 [1.85–2.15]	1.97 [1.83–2.05]	2.15 [2.12–2.17]	<0.0001
**NLR, median [Q1–Q3]**	3.58 [2.41–5.67]	2.86 [2.2–4.34]	5.9 [5.31–8.18]	<0.0001
**PLR, median [Q1–Q3]**	140.59 [107.4–208.39]	129.96 [103.17–174.17]	208.39 [139.8–269.79]	<0.0001
**SII, median [Q1–Q3]**	823.59 [436.91–1277.02]	641.99 [410.26–999.93]	1294.63 [963.3–1907.42]	<0.0001
**Type of AVF**				
**RC-AVF, no. (%)**	64 (51.2%)	47 (53.41%)	17 (45.95%)	0.44 (0.74; 0.34–1.60)
**Radial artery diameter, median [Q1–Q3]**	2.4 [2.08–3]	2.8 [2.3–3.25]	2.05 [1.9–2.2]	<0.0001
**Cephalic vein diameter, median [Q1–Q3]**	2.8 [2.1–4.22]	3.3 [2.5–4.6]	2.1 [1.9–2.3]	<0.0001
**BC-AVF, no. (%)**	61 (48.8%)	41 (46.59%)	20 (54.05%)	0.44 (1.34; 0.62–2.91)
**Brachial artery diameter, median [Q1–Q3]**	3.5 [2.5–4.5]	3.8 [3.1–5]	2.5 [2.32–2.67]	<0.0001
**Cephalic vein diameter, median [Q1–Q3]**	3.4 [2.1–5.8]	4.2 [3.4–6.5]	2.1 [1.8–2.32]	<0.0001
**Outcomes**				
**Early thrombosis, no. (%)**	22 (17.6%)	-	22 (43.24%)	0.0001
**Mortality, no. (%)**	10 (8.0%)	3 (3.41%)	7 (18.92%)	0.008 (6.61; 1.60–27.21)

AH = arterial hypertension; AF = atrial fibrillation; CHF = chronic heart failure; IHD = ischemic heart disease; MI = myocardial infarction; T2D = type 2 diabetes; CVA = cerebrovascular accident; PAD = peripheral arterial disease; PLT = total platelet count; NLR = neutrophil to lymphocyte ratio; PLR = platelet to lymphocyte ratio; SII = systemic inflammatory index; PNI = prognostic nutritional index; CRP = C-reactive protein; RC-AVF = radio-cephalic arteriovenous fistula; and BC-AVF = brachio-cephalic arteriovenous fistula.

**Table 2:** Outcomes of all patients included in the analysis and of the two sub-groups evaluated according to AVF type.

Outcome	All Patients *n* = 125	RC-AVF *n* = 64	BC-AVF *n* = 61	*p*-Value
Six-week maturation, no. (%)	88 (70.4%)	47 (73.43%)	41 (67.21%)	0.44
Early thrombosis, no. (%)	22 (17.6%)	9 (14.06%)	13 (61.31%)	0.29
Mortality, no. (%)	10 (8%)	4 (6.25%)	6 (9.83%)	0.46
Overall maturation, no. (%)	109 (87.2%)	51 (79.68%)	58 (95.08%)	0.01

RC-AVF = radio-cephalic arteriovenous fistula; BC-AVF = brachio-cephalic arteriovenous fistula.

**Table 3:** ROC curves, optimal cut-off values, AUCs, and predictive accuracies of the NLR, PLR, SII, and CRP inflammatory markers, Ca-P product, the PNI, and vessel diameters.

Variables		Cut-Off	AUC	Std. Error	95% CI	Sensitivity	Specificity	*p*-Value
	**Non-Maturation**							
**NLR**		4.90	0.856	0.039	0.780–0.932	81.1%	84.1%	<0.0001
**PLR**		172.29	0.740	0.051	0.639–0.841	70.3%	73.9%	<0.0001
**SII**		954.54	0.802	0.044	0.716–0.888	78.4%	72.7%	<0.0001
**PNI**		40.59	0.852	0.036	0.780–0.923	80.7%	81.1%	<0.0001
**Ca-P product**		47.36	0.859	0.038	0.784–0.934	81.1%	80.7%	<0.0001
**CRP**		2.07	0.785	0.043	0.700–0.871	83.8%	73.9%	<0.0001
**RC-AVF**	**RA diameter**	2.25	0.869	0.044	0.783–0.956	78.7%	88.2%	<0.0001
	**CV diameter**	2.55	0.866	0.044	0.779–0.953	72.3%	99.05%	<0.0001
**BC-AVF**	**BA diameter**	2.95	0.841	0.050	0.742–0.940	82.9%	80%	<0.0001
	**CV diameter**	2.70	0.894	0.043	0.810–0.978	85.4%	90%	<0.0001
	**Early Thrombosis**							
**NLR**		4.90	0.780	0.050	0.681–0.878	77.3%	73.8%	<0.0001
**PLR**		181.72	0.739	0.066	0.611–0.868	72.7%	71.8%	<0.0001
**SII**		859.22	0.736	0.056	0.626–0.845	81.8%	61.2%	0.001
**PNI**		38.65	0.839	0.038	0.766–0.913	78.6%	81.8%	<0.0001
**Ca-P product**		49.67	0.777	0.054	0.671–0.883	72.7%	80.6%	<0.0001
**CRP**		2.07	0.785	0.042	0.702–0.869	86.4%	66%	<0.0001
**RC-AVF**	**RA diameter**	2.35	0.826	0.052	0.725–0.927	61.8%	100%	0.002
	**CV diameter**	2.35	0.857	0.049	0.761–0.952	74.5%	88.9%	0.001
**BC-AVF**	**BA diameter**	2.95	0.784	0.065	0.621–0.876	70.8%	69.2%	0.006
	**CV diameter**	2.70	0.780	0.058	0.667–0.894	75%	99.3%	0.002
	**Mortality**							
**NLR**		5.83	0.846	0.059	0.730–0.962	80%	83.5%	<0.0001
**PLR**		212.89	0.817	0.053	0.713–0.922	80%	80.9%	0.001
**SII**		949.71	0.777	0.061	0.656–0.897	90%	60.9%	0.004
**PNI**		33.20	0.904	0.052	0.803–1.000	91.3%	80%	0.01
**Ca-P product**		41.36	0.714	0.075	0.566–0.862	90%	58.3%	0.02
**CRP**		2.15	0.785	0.081	0.626–0.943	70%	82.6%	0.001
**RC-AVF**	**RA diameter**	2.35	0.771	0.071	0.611–0.931	56.7%	100%	0.07
	**CV diameter**	2.15	0.902	0.044	0.815–0.989	78.3%	100%	0.007
**BC-AVF**	**BA diameter**	2.70	0.786	0.066	0.656–0.917	70.9%	83.3%	0.02
	**CV diameter**	2.45	0.792	0.059	0.677–0.907	70.9%	83.3%	0.01

NLR = neutrophil to lymphocyte ratio; PLR = platelet to lymphocyte ratio; SII = systemic inflammatory index; PNI = prognostic nutritional index; CRP = C-reactive protein; RC-AVF = radio-cephalic arteriovenous fistula; BC-AVF = brachio-cephalic arteriovenous fistula; RA = radial artery; BA = brachial artery; and CV = cephalic vein.

**Table 4:** Univariate analysis of the NLR, PLR, SII, CRP, Ca-P product, PNI, vessel diameters, and all adverse event occurrences during the studied period for all patients.

	Non-Maturation	Early Thrombosis	Mortality
**Low NLR vs. high NLR**	74/81 (91.36%) vs. 14/44 (31.88%) *p* < 0.0001 OR: 22.65 CI: (8.32–61.67)	5/81 (6.17%) vs. 17/44 (38.64%) *p* < 0.0001 OR: 9.57 CI: (3.21–28.45)	2/97 (2.06%) vs. 8/28 (28.57%) *p* = 0.0004 OR: 19 CI: (3.74–96.27)
**Low PLR vs. high PLR**	65/76 (85.55%) vs. 23/49 (46.94%) *p* < 0.0001 OR: 9.66 CI: (3.88–24.07)	6/80 (7.50%) vs. 16/45 (35.55%) *p* = 0.0003 OR: 6.80 CI: (2.42–19.09)	2/95 (2.10%) vs. 8/30 (26.67%) *p* = 0.0006 OR: 16.90 CI: (3.35–85.24)
**Low SII vs. high SII**	64/72 (88.89%) vs. 24/53 (44.28%) *p* < 0.0001 OR: 9.66 CI: (3.88–24.07)	4/67 (5.97%) vs. 18/58 (31.03%) *p* = 0.0009 OR: 7.08 CI: (2.23–22.46)	1/71 (1.40%) vs. 9/54 (16.67%) *p* = 0.01 OR: 14.0 CI: (1.71–114.29)
**Low PNI vs. high PNI**	16/46 (34.78%) vs. 72/79 (91.14%) *p* < 0.0001 OR: 0.05 CI: (0.01–0.13)	15/40 (37.50%) vs. 7/85 (8.23%) *p* = 0.0002 OR: 0.14 CI: (0.05–0.40)	8/19 (42.11%) vs. 2/106 (1.89%) *p* < 0.0001 OR: 0.02 CI: (0.005–0.14)
**Low Ca-P product vs. High Ca-P product**	69/78 (88.46%) vs. 19/47 (40.43%) *p* < 0.0001 OR: 11.29 CI: (4.56–27.97)	6/89 (6.74%) vs. 16/36 (44.44%) *p* < 0.0001 OR: 11.06 CI: (3.84–31.86)	1/69 (1.47%) vs. 9/57 (15.79%) *p* = 0.01 OR: 12.75 CI: (1.56–103.99)
**Low CRP vs. high CRP**	63/71 (88.73%) vs. 25/54 (46.30%) *p* < 0.0001 OR: 9.13 CI: (3.67–22.68)	3/71 (4.23%) vs. 19/54 (35.19%) *p* = 0.0001 OR: 12.30 CI: (3.40–44.43)	3/94 (3.19%) vs. 7/31 (22.58%) *p* = 0.002 OR: 8.84 CI: (2.12–36.79)
**RC-AVF**	**Non-Maturation**	**Early Thrombosis**	**Mortality**
**Low RA diameter vs. high RA diameter**	10/25 (40%) vs. 37/39 (94.87%) *p* = 0.0009 OR: 14.6 CI: (3.02–70.60)	8/30 (26.67%) vs. 1/34 (2.94%) *p* = 0.02 OR: 0.08 CI: (0.009–0.71)	4/30 (13.33%) vs. 0/34 (0%) *p* = 0.10 OR: 0.08 CI: (0.004–1.65)
**Low CV diameter vs. high CV diameter**	13/29 (44.82%) vs. 34/35 (97.14%) *p* = 0.0001 OR: 27.75 CI: (5.42–141.98)	8/22 (36.36%) vs. 1/42 (2.38%) *p* = 0.004 OR: 0.04 CI: (0.004–0.37)	4/17 (23.52%) vs. 0/47 (0%) *p* = 0.02 OR: 0.03 CI: (0.001–0.62)
**BC-AVF**	**Non-Maturation**	**Early Thrombosis**	**Mortality**
**Low BA diameter vs. high BA diameter**	7/23 (30.43%) vs. 34/38 (89.47%) *p* < 0.0001 OR: 19.42 CI: (4.96–76.05)	9/23 (39.13%) vs. 4/38 (10.52%) *p* = 0.01 OR: 0.18 CI: (0.04–0.69)	5/21 (23.80%) vs. 1/40 (2.50%) *p* = 0.02 OR: 0.08 CI: (0.008–0.75)
**Low CV diameter vs. high CV diameter**	6/24 (40%) vs. 35/37 (94.59%) *p* < 0.0001 OR: 52.5 CI: (9.60–286.89)	11/24 (45.83%) vs. 3/37 (8.10%) *p* = 0.001 OR: 0.10 CI: (0.02–0.43)	5/21 (23.80%) vs. 1/40 (2.50%) *p* = 0.02 OR: 0.08 CI: (0.008–0.75)

NLR = neutrophil to lymphocyte ratio; PLR = platelet to lymphocyte ratio; SII = systemic inflammatory index; PNI = prognostic nutritional index; CRP = C-reactive protein; RC-AVF = radio-cephalic arteriovenous fistula; BC-AVF = brachio-cephalic arteriovenous fistula; RA = radial artery; BA = brachial artery; and CV = cephalic vein.

**Table 5:** Multivariate analysis of the new adverse events that occurred during the study period.

		Non-Maturation			Early Thrombosis			Mortality		
		OR	95% CI	*p*-Value	OR	95% CI	*p*-Value	OR	95% CI	*p*-Value
**CHF**		4.38	3.88–24.07	<0.001	3.71	1.41–9.71	0.008	1.11	0.29–4.18	0.87
**MI**		1.52	0.70–3.30	0.28	1.67	0.66–4.22	0.27	1.30	0.35–4.73	0.69
**T2D**		5.63	2.43–13.06	<0.001	3.82	1.43–10.21	0.008	0.93	0.24–3.47	0.91
**Tobacco**		1.72	0.77–3.80	0.17	1.45	0.54–3.61	0.48	0.45	0.09–2.22	0.32
**RC-AVF**	**High RA diameter**	0.03	0.007–0.18	<0.001	0.05	0.006–0.48	0.009	0.19	0.01–1.97	0.16
	**High CV diameter**	0.02	0.003–0.19	<0.001	0.04	0.005–0.37	0.004	0.04	0.009–0.75	0.04
**BC-AVF**	**High BA diameter**	0.05	0.01–0.20	<0.001	0.18	0.04–0.69	0.01	0.08	0.009–0.75	0.02
	**High CV diameter**	0.01	0.003–0.10	<0.001	0.02	0.003–0.23	0.001	0.08	0.009–0.75	0.02
**High NLR**		22.65	8.32–61.67	<0.001	9.57	3.21–28.45	<0.001	19.0	3.75–96.27	<0.001
**High PLR**		6.68	2.85–15.63	<0.001	6.80	2.42–19.09	<0.001	16.90	3.35–85.24	<0.001
**High SII**		9.66	3.88–24.07	<0.001	7.08	2.23–22.46	<0.001	14.0	1.71–114.28	0.01
**High PNI**		0.05	0.02–0.14	<0.001	0.15	0.05–0.40	<0.001	0.02	0.005–0.14	<0.001
**High Ca-P Product**		17.89	6.73–47.60	<0.001	11.06	3.84–31.86	<0.001	12.56	1.54–102.48	0.01
**High CRP**		14.60	5.39–39.49	<0.001	12.30	3.40–44.43	<0.001	8.84	2.12–36.79	0.003

CHF = chronic heart failure; MI = myocardial infarction; T2D = type 2 diabetes; NLR = neutrophil to lymphocyte ratio; PLR = platelet to lymphocyte ratio; SII = systemic inflammatory index; PNI = prognostic nutritional index; CRP = C-reactive protein; RC-AVF = radio-cephalic arteriovenous fistula; BC-AVF = brachio-cephalic arteriovenous fistula; RA = radial artery; BA = brachial artery; and CV = cephalic vein.
